# The impact of gut microbial signals on hematopoietic stem cells and the bone marrow microenvironment

**DOI:** 10.3389/fimmu.2024.1338178

**Published:** 2024-02-13

**Authors:** Xiru Liu, Hao Zhang, Guolin Shi, Xinmin Zheng, Jing Chang, Quande Lin, Zhenhao Tian, Hui Yang

**Affiliations:** ^1^ School of Life Sciences, Northwestern Polytechnical University, Xi'an, China; ^2^ Engineering Research Center of Chinese Ministry of Education for Biological Diagnosis, Treatment and Protection Technology and Equipment, Xi'an, China; ^3^ Research Center of Special Environmental Biomechanics & Medical Engineering, Northwestern Polytechnical University, Xi'an, China; ^4^ Medical Service, The Affiliated Cancer Hospital of Zhengzhou University & Henan Cancer Hospital, Zhengzhou, China

**Keywords:** bone marrow microenvironment, gut microbiota, hematopoietic stem cells, hematologic malignancies, immune cells, broad-spectrum antibiotics

## Abstract

Hematopoietic stem cells (HSCs) undergo self-renewal and differentiation in the bone marrow, which is tightly regulated by cues from the microenvironment. The gut microbiota, a dynamic community residing on the mucosal surface of vertebrates, plays a crucial role in maintaining host health. Recent evidence suggests that the gut microbiota influences HSCs differentiation by modulating the bone marrow microenvironment through microbial products. This paper comprehensively analyzes the impact of the gut microbiota on hematopoiesis and its effect on HSCs fate and differentiation by modifying the bone marrow microenvironment, including mechanical properties, inflammatory signals, bone marrow stromal cells, and metabolites. Furthermore, we discuss the involvement of the gut microbiota in the development of hematologic malignancies, such as leukemia, multiple myeloma, and lymphoma.

## 1 Introduction

Hematopoiesis is a complex and highly regulated biological process that produces a variety of blood cells and specialized immune cells necessary for multiple bodily functions. Additionally, hematopoiesis plays a critical role in immune regulation, including oxygen transportation, hemostasis, and both innate and adaptive immunity (1, 2). It is widely accepted that hematopoietic stem cells (HSCs) are at the top of the hematopoietic hierarchy. HSCs are initially found in the yolk sac of the human embryo at two weeks of gestation. As the fetus develops, HSCs migrate to hematopoietic organs, primarily the bone marrow, where they persist throughout life (3). HSCs possess two distinct characteristics: self-renewal, which allows them to maintain their own population, and multilineage differentiation, which enables them to differentiate into various types of hematopoietic cells. This process is tightly regulated by signals from the bone marrow microenvironment, ensuring the balance of the blood system and overall body functions (4). Disruption of the bone marrow microenvironment, including stromal cells such as fibroblasts, pericytes, and mesenchymal stem cells, can impact the activity and function of HSCs.

The human body is inhabited by diverse microbiota, which can be found in various parts such as the skin, oral cavity, alveoli, gastrointestinal tract, and genitourinary tract (5). Among these, the gastrointestinal tract harbors the highest number of microorganisms, accounting for approximately 78% of the total microorganisms in the human body. This collection of microorganisms is known as the gut microbiota (6, 7). Comprising bacteria, fungi, viruses, and protozoa, the gut microbiota plays a critical role in regulating metabolism and immune homeostasis (8). Gut Microbiota, also known as the ‘second largest genome’ of human beings, is the most complex micro-ecosystem in the human body. It maintains a symbiotic relationship with the host through various processes such as the fermentation of dietary fiber, defense against pathogens, and synthesis of metabolites like short-chain fatty acids (SCFAs). The gut microbial homeostasis also influences the maturation and function of the body’s immune system. For instance, SCFAs play an immunomodulatory role in gut homeostasis for the host (9). Studies have demonstrated that SCFAs can diffuse from the gut into the bloodstream and reach the bone marrow, promoting hematopoiesis (10). Germ-free mice, which lack microbiota, exhibit immune system defects and alterations in the hematopoietic environment, including reduced hematopoietic progenitor cells in the basal metabolism (11, 12). Therefore, manipulating the gut microbiota to enhance the activity and differentiation of HSCs holds promise as a potential therapeutic intervention for hematopoietic diseases. In this review, we focus on the impact of the gut microbiota on the fate differentiation of HSCs and its influence on the bone marrow microenvironment. Additionally, we discuss the role of intervening in the gut microbiota in the development of hematological malignancies.

## 2 HSCs and bone marrow microenvironment

### 2.1 The fate of HSCs

**Figure 1:**
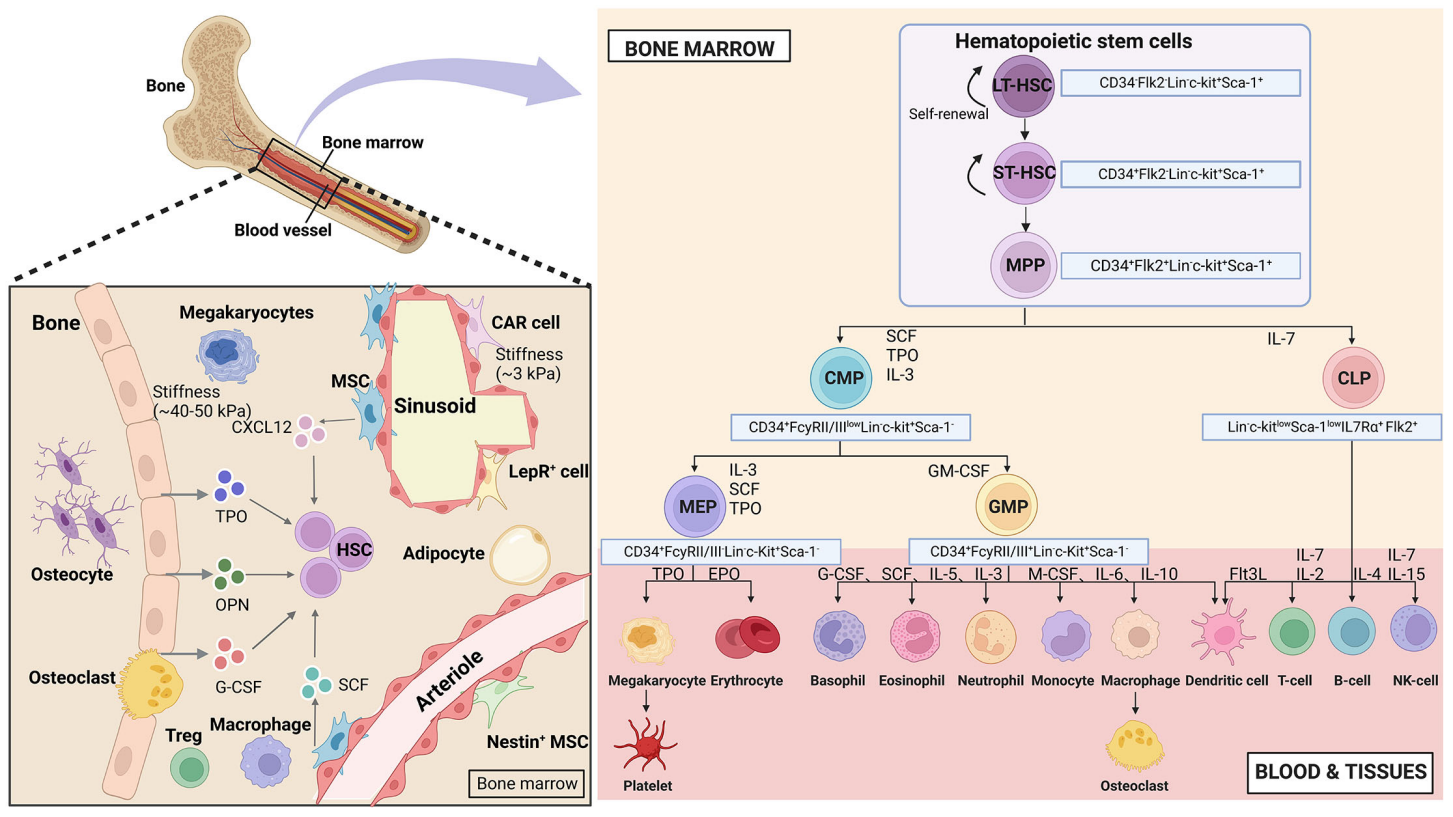
Hematopoietic hierarchical models and the bone marrow microenvironment. Top HSCs consist of diverse populations that possess the ability to self-renew and differentiate in multiple directions. This process involves a binary branch point that separates the myeloid and lymphoid lineages. In the middle and terminal stages, oligopotent cells further divide into distinct unipotent cells through discrete differentiation stages. The bone marrow niches consist of various stromal cells and cytokines, with the areas near the endosteal niche being stiffer (40-50 kPa) and the perivascular areas being softer (3 kPa). (Created with BioRender.com, academic licenses have been granted).

HSCs are the apex of the hematopoietic system, possessing the remarkable ability to self-renew and differentiate. This capacity allows them to sustain blood regeneration and meet the increased demand for immune cells during periods of stress (4). In humans, HSCs are characterized by surface markers CD34, c-Kit (CD117), and CD90. In mice, the purification protocol for HSCs involves identifying and expressing (Lin^-^, c-Kit^+^, and Sca-1^+^) in conjunction with the signaling lymphocyte activation molecule family of protein receptors (CD150 and CD48) (13). To visualize the relationship between HSCs and their progeny more clearly, a hierarchical model called the HSCs tree has been established (14). This model categorizes HSCs into two subpopulations based on CD34 expression: CD34^-^ long-term-HSCs and CD34^+^ short-term-HSCs (15). Long-term-HSCs remain in a quiescent state and have the potential for long-term reconstitution (lasting beyond 3-4 months), while short-term-HSCs exhibit a shorter reconstitution ability (typically less than one month). Long-term-HSCs give rise to short-term-HSCs, which differentiate into multipotent progenitors (MPPs). These cells are hematopoietic stem and progenitor cells (HSPCs) and share the expression of Lin^-^, Sca-1^+^, and c-Kit^+^ markers in mice. MPPs differentiate into common myeloid progenitors (CMPs, with potential for myeloid, erythroid, and megakaryocyte lineages) and common lymphoid progenitors (CLPs, with potential for lymphoid lineages). CMPs further differentiate into granulocyte-macrophage progenitors (GMPs) and megakaryocyte-erythrocyte progenitors (MEPs). CLPs give rise to T, B, NK, and dendritic cells. GMPs differentiate into granulocytes and monocytes, while MEPs produce erythrocytes and megakaryocytes (16, 17). All these populations form a hierarchical and balanced structure resembling a tree (**Figure 1**).

### 2.2 Hematopoietic ecological niche - bone marrow microenvironment

*In vivo*, stem cells reside in ecological niches, which are fundamental microenvironments. These niches integrate various factors to determine the fate of the stem cells. During embryonic development, HSCs niches are present in different tissues at different stages. In vertebrates, the hematopoietic system originates from the yolk sac in early embryonic development. Hematopoietic differentiation starts in the endothelium of the aorta-gonad-mesonephros region^16^. After this stage, hematopoietic cells migrate through the bloodstream to the liver and spleen, eventually settling and establishing residence within the bone marrow (18). In 1978, Schofield proposed the concept of the HSCs niche in the bone marrow (19). These niches are complex structures consisting of hematopoietic and non-hematopoietic cells. bone marrow niches are multidimensional complex systems that involve mechanical properties (stiffness and surface tension), cellular composition (endothelial cells, mesenchymal stromal cells, and osteoblasts), and biochemical signals (oxygen concentration, hormones, growth factors, chemokines, and cytokines) that regulate the fate of HSCs (**Figure 1**).

In the bone marrow, HSCs are constantly in a hypoxic state, regardless of their ecological niche. Each bone marrow ecological niche has unique physical spatial properties. The stiffness of the matrix in the bone marrow niche varies due to its inhomogeneity. Osteoclasts and osteoblast progenitors are densely located in the endothelial ecotone and have a relative stiffness of approximately 35-40 kPa. On the other hand, the vascular niche, which is highly populated by adipocytes and endothelial cells has a relative stiffness of roughly 3 kPa. The central bone marrow is the softest, with a matrix stiffness of approximately 0.3 kPa (20–22).

The bone marrow niche is composed of various stromal cells. Osteoblasts, which are responsible for bone formation, are located in the endosteal niche along with HSCs. These were the first group of bone marrow cells discovered to play a role in regulating HSCs (23). Another type of stromal cell found in the bone marrow is mesenchymal stem cells (MSCs), which have the ability to self-renew and differentiate into adipocytes and chondrocytes. MSCs are distributed around blood vessels and contribute to the formation of the hematopoietic niche (24). The perivascular stromal cells surrounding the blood vessels consist of various cell types, including endothelial cells, CXCL12-abundant reticular (CAR) cells, leptin receptor-positive (Lepr^+^) perivascular stromal cells, Nestin^+^ MSCs, regulatory T cells (Tregs), and megakaryocytes. All of these cell types are involved in maintaining HSCs (25).

Several biochemical signals, including cytokines, secreted by stromal cells have an impact on the homeostasis of HSCs. The inner layer of blood vessels contains endothelial cells that provide nutrients and oxygen to various organs, including the bone marrow (26). HSCs are positioned in close proximity to endothelial cells, and these cells regulate HSCs by producing stem cell factor (SCF) and Jag1. CAR cells, which co-express LepR^+^ and Nes-GFP^+^, are primarily located around the blood sinuses. Perivascular stromal cells found on small arterioles, expressing NG2^+^ and Nes-GFP^+^, also secrete CXCL12, which controls the mobilization pool of HSCs. However, in the vicinity of the blood sinuses, CXCL12 secreted by perivascular stromal cells (CAR, LepR^+^, Nes^-^GFP^+^ cells) contributes to the retention of HSCs. Non-myelinating Schwann cells have the ability to activate latent TGFβ, which is crucial for maintaining HSCs. Additionally, mature hematopoietic cells play a role in regulating HSCs function. CD169^+^ bone marrow macrophages enhance HSCs retention by increasing CXCL12 production from CAR cells, while megakaryocytes maintain HSCs in a quiescent state through the secretion of CXCL4, TGFβ, and thrombopoietin (TPO) (4).

### 2.3 Bone marrow microenvironment regulates the fate of HSCs

HSCs fate differentiation derives primarily from regulating bone marrow microenvironmental, including mechanical properties, inflammatory factors, and other stromal cells in the bone marrow.

#### 2.3.1 Mechanical properties

HSCs lineage differentiation is heavily influenced by matrix stiffness (27). The fate of HSCs is regulated by physical factors and spatial properties in the bone marrow. Physical properties include matrix stiffness and elasticity, while spatial properties refer to surface topography and dimensionality (28). The variations in stiffness determine whether HSCs will undergo self-renewal or differentiation. HSCs exhibit greater adhesion and motility on hard surfaces compared to soft surfaces, suggesting that long-term HSCs are found in stiffer endothelial niches (29). Another study demonstrated that substrate stiffness also impacts the differentiation of HSCs. Stiffer substrates promote the differentiation of HSCs into primitive myeloid progenitors, whereas softer substrates encourage differentiation towards the erythroid lineage (27). To better maintain and expand HSCs, three-dimensional (3D) scaffolds that mimic the bone marrow environment have shown to be more effective than conventional two-dimensional (2D) culture systems (30). Similarly, MSCs have a greater positive impact on HSPC proliferation in 3D polyethylene glycol (PEG) co-culture systems compared to standard 2D culture systems (31). Combining stromal cells with biological scaffolds provides a more accurate representation of the bone marrow microenvironment. Our research group investigated the impact of matrix dimensions on HSC regulation. In the 3D system, HPC formed “3D macrophage” clusters, which were not observed in the 2D culture. Single-cell sequencing analysis revealed that the 3D matrix enhanced communication between these 3D macrophages and other hematopoietic clusters, resulting in a significant increase in Lin^-^, c-kit^+^ cells (32). Furthermore, several studies have demonstrated that nanomorphology can influence the behavior of HSCs (33).

#### 2.3.2 Inflammatory factors

Young HSCs are found in specialized ecological niches within the bone marrow microenvironment. In these niches, HSCs remain dormant and protected from external stresses. During homeostasis, there are minor subpopulations of HSCs called MPP2 and MPP3, which favor erythroid/megakaryocyte and granulocyte/macrophage differentiation, respectively. The majority subpopulation, MPP4, favors lymphoid differentiation. However, when inflammation occurs, HSCs become activated and their self-renewal capacity is reduced. This is due to an increase in MPP2/MPP3 and a shift in MPP4 output towards the myeloid spectrum, leading to the formation of GMP (34). This activation process is influenced by various inflammatory cytokines that act at the early hematopoietic level. For example, IL-1β (35), Granulocyte colony-stimulating factor (G-CSF) (36), and type I and type II interferon (IFN) can promote HSC proliferation and myeloid differentiation (37). These cytokines also regulate the expression of key transcription factors (TFs) involved in myeloid commitment, such as Pu.1 (38). TNFα, IL-1β, and M-CSF can induce myeloid differentiation by upregulating Pu.1 (39), while IFN stimulates the expression of Batf2 and Cebpβ, which promote myeloid spectrum differentiation (40). Additionally, cytokines like IL-6 primarily affect MPP4, redirecting its output from lymphatic to myeloid lineage (41). Cytokines such as G-CSF regulate the formation of GMP clusters (34). The combined effects of these inflammatory signaling pathways result in changes to the differentiation profile of HSCs and HSPCs, temporarily meeting the organism’s needs and then restoring homeostasis *in vivo*.

#### 2.3.3 Other matrix cells

The bone marrow microenvironment consists of various cell types that have specific functions (42). These cells directly or indirectly support the maintenance and regulation of HSCs (4). MSCs release factors like CXCL12, SCF, and IL-7 (25), which play a crucial role in regulating HSCs (43). Bone lineage cells are necessary for the production of lymphoid tissue. Osteoblasts produce factors like osteopontin and G-CSF, as well as release thrombopoietin (TPO), which regulates HSCs production (44, 45). Adipogenesis is an emergency response that enhances hematopoiesis when blood cells decrease (46). Bone marrow adipocytes synthesize adiponectin, which promotes HSCs regeneration after irradiation by stimulating their proliferation. Adipocytes also secrete various factors that influence the function of HSCs and stromal cells in the bone marrow (47). Aging and obesity lead to the accumulation of adipocytes in the bone marrow cavity, inhibiting HSCs activity and impairing hematopoietic regeneration (48, 49). Endothelial cells may regulate HSCs maintenance and the activity of perivascular cells. Macrophages are directly involved in HSCs maintenance and indirectly control HSCs retention through other microenvironment cells (50). Megakaryocytes promote HSCs quiescence through feedback loops (51). T cells and neutrophils interact with HSCs directly or through other immune cells and stromal cells to regulate their behavior. Granulocytes also interact directly with HSCs or control their behavior through other immune cells and stromal cells (52). Despite numerous studies, the regulation of HSCs populations in the bone marrow microenvironment remains highly complex and elusive.

## 3 The correlation between gut microbiota and hematopoiesis

### 3.1 Gut microbiota depletion and antibiotics affect hematopoiesis

**Figure 2:**
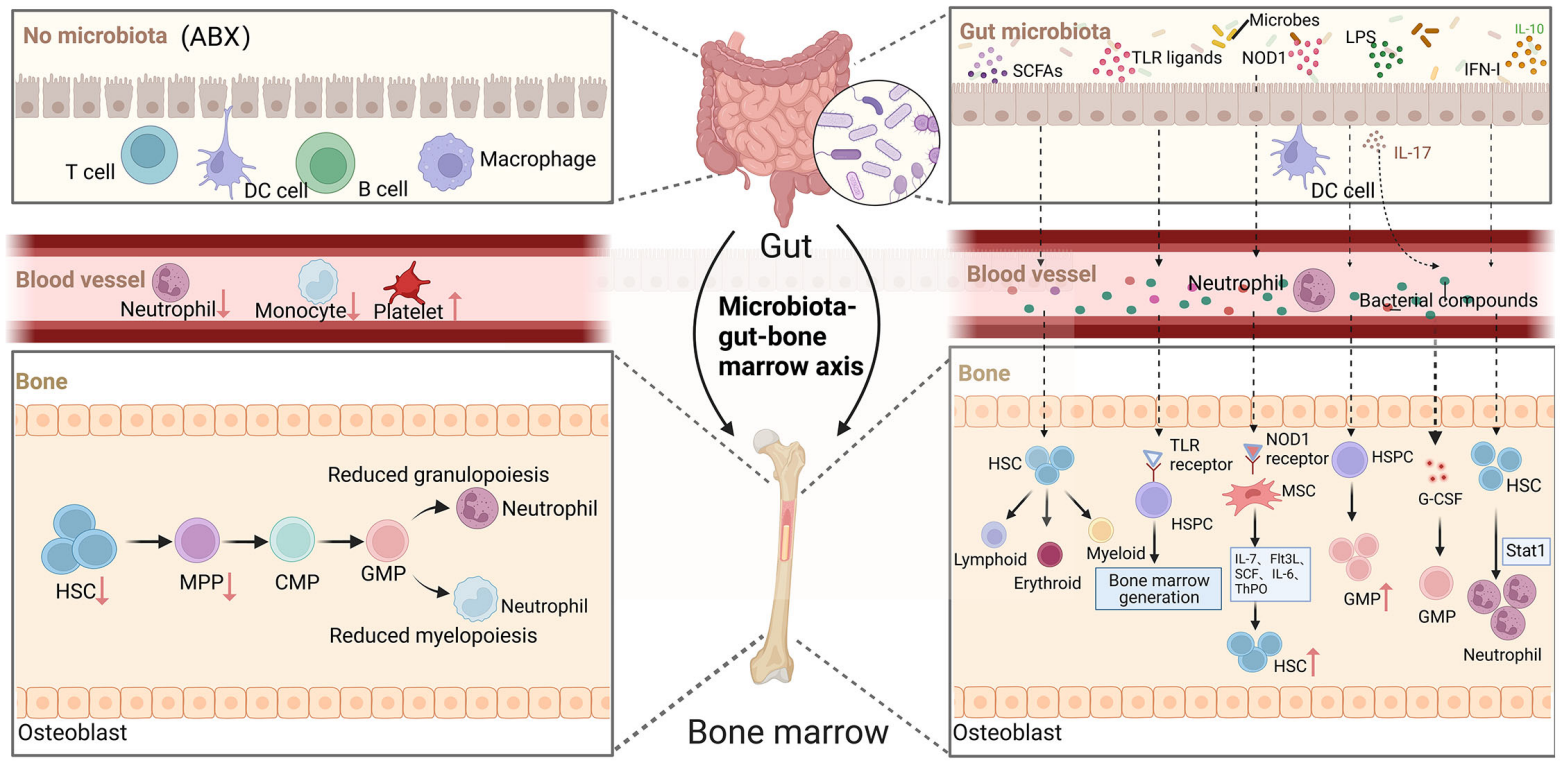
The role of gut microorganisms in hematopoiesis. Depletion of gut microbiota leads to defects in the immune cell population in the blood, such as neutrophils and monocytes, which in turn reduces HSCs and decreases myeloid and lymphoid differentiation. The complex gut microbiota and their metabolites enter the bone marrow via the bloodstream and have a significant impact on hematopoiesis in multiple ways. (Created with BioRender.com, academic licenses have been granted).

The complex gut microbial community has both local and systemic effects on host immunity, including the regulation of immune cell development and maturation (53, 54). In a steady-state environment, signals from the gut microbiota are crucial for maintaining normal hematopoiesis (55). In clinical settings, some patients experience disruption of the gut microbiota following antibiotic treatment, which is accompanied by hematological abnormalities such as neutropenia, anemia, thrombocytopenia, and leukopenia (56, 57). Mouse experiments have also shown that oral administration of antibiotics depletes the gut microbiota and inhibits hematopoietic function. Mice treated with broad-spectrum antibiotics exhibit anemia, increased platelets in peripheral blood, and decreased white blood cells and lymphocytes. However, when HSPCs are directly co-cultured with antibiotics, no decrease in progenitor cell activity is observed. The impact of antibiotic treatment on bone marrow cells in germ-free mice is not further observed (11). In germ-free mice, the absence of microbial signals leads to a decrease in HSCs, MPPs, and bone marrow progenitor cell numbers. NOD1 ligand administration can enhance hematopoietic cytokines, which contributes to the expansion of the HSPCs pool and hematopoietic maintenance (58). Co-housing germ-free mice with conventionally raised mice modifies the diversity of the gut microbiota, increases myeloid cell production and T cell activation, promotes HSCs reconstruction, and alters the expression of hematopoietic genes (59). These studies above suggesting that antibiotics disrupt hematopoietic function by affecting the balance and diversity of the gut microbiota (**Figure 2**).

### 3.2 Gut microbiota influenced hematopoiesis beginning in the embryonic period

Microbial colonization initiates with the transmission of microorganisms from the mother to the infant during birth. During early life, these microbial signals play a crucial role in shaping the development of hematopoietic stem and HSPCs in the bone marrow. Metabolites produced by the maternal microbiota can cross the placenta and be transferred to the fetus, suggesting that the microbiota may have a potential impact on fetal HSPC development (60). In zebrafish, it has been discovered that the microbiota regulates the development of HSCs and HSPCs by mediating inflammatory signals in their niche. Maintaining a balanced microbiota is crucial for the proper development and homeostasis of HSPCs during embryonic life. Specific bacterial species have been found to influence the formation and differentiation of HSPCs by modulating the local production of inflammatory cytokines near these cells (61). Metabolites containing microbial molecules, such as short-chain fatty acids (SCFAs), can be transmitted to the fetus through breast milk, where they regulate the immune system and inflammatory responses in the offspring (62). Additionally, microbe-associated molecular patterns (MAMPs) can enter the bloodstream from the gut and reach the bone marrow, where they play a role in regulating the proliferation and differentiation of HSCs, MPPs, and their descendants (63). Following birth, the fetus can inherit HSCs and other hematopoietic progenitor cells from the mother through umbilical cord blood. Dysbiosis of the maternal microbiota can impact the neonatal bone marrow environment by altering the behavior of these maternal-derived progenitor cells. Microbial signals have the ability to regulate the immune system of children at various stages of development (64).

## 4 Gut microbiota influences HSCs fate by modulating the bone marrow microenvironment

**Figure 3:**
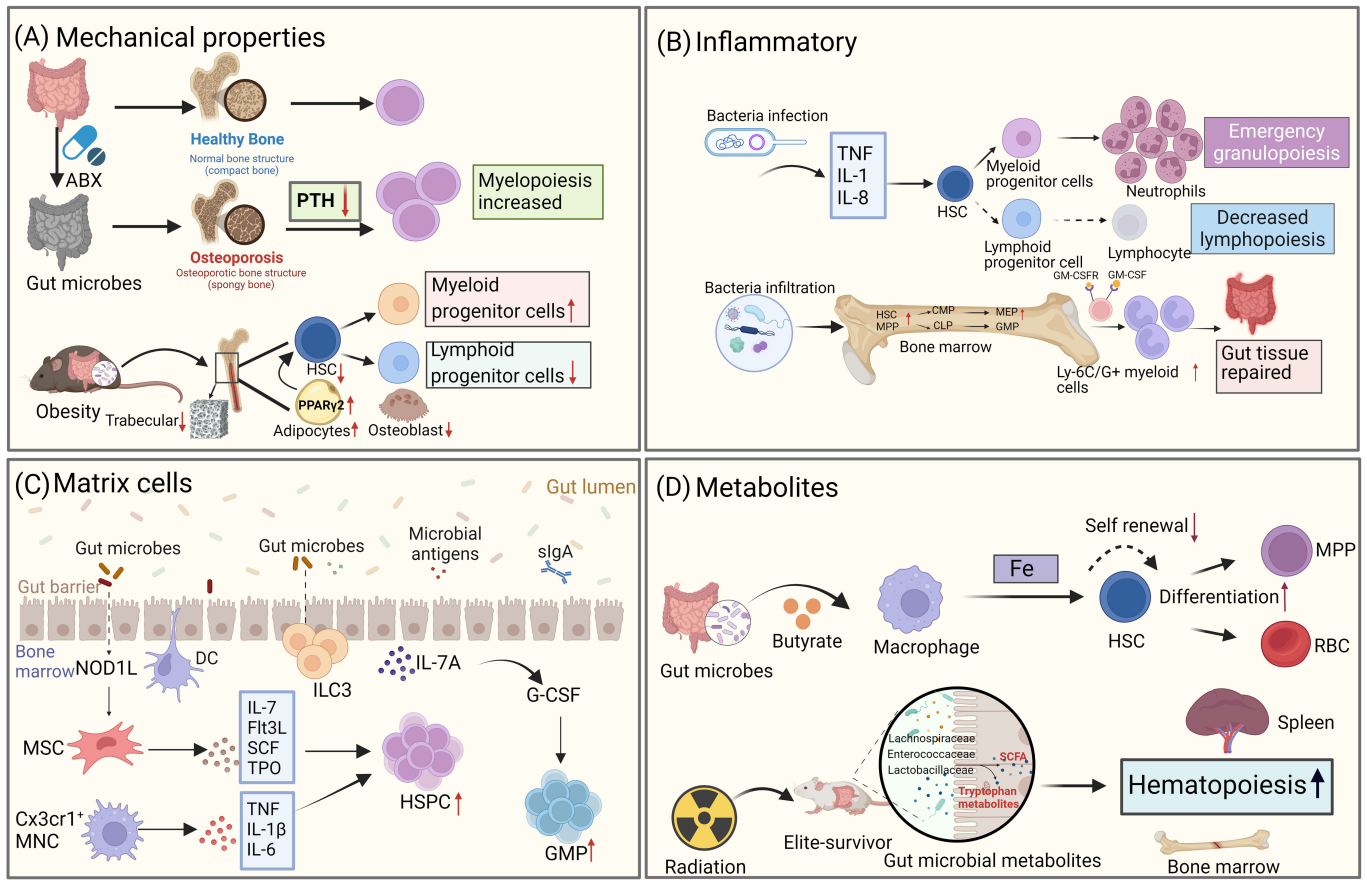
Gut microbiota influences the fate differentiation of HSCs through the bone marrow microenvironment. **(A)** Gut microbiota alters the mechanical properties of the bone marrow, which in turn affects the fate of HSCs. Disruption of the gut microbiome through antibiotic treatment leads to osteoporosis, resulting in changes to bone strength and mechanical properties, as well as decreased levels of parathyroid hormone (PTH). This, in turn, affects the bias of HSPCs towards the bone marrow. In high-fat obese mice, gut microbiota alterations also impact the trabecular number of the bone marrow, leading to a reduction in the number of LSK cells. **(B)** Gut microbiota regulates hematopoietic fate by influencing inflammatory signaling. During infection, microbes trigger the production of pro-inflammatory factors, which drive HSCs to undergo emergency myeloid cell regeneration. Additionally, microbes can differentiate into intestinal repair cells during acute intestinal inflammation by expanding HSCs. **(C)** Gut microbiota influences HSCs fate through stromal cells in the bone marrow. NOD1 ligands from the microbial community contribute to the expansion of the HSPC pool and the maintenance of homeostatic hematopoiesis by inducing the production of various hematopoietic cytokines (IL-7, Flt3L, SCF, TPO, and IL-6) in MSCs. CX3CR1^+^ monocytes capture bacterial DNA and produce TNF-α, IL-1β, and IL-6, which control HSPC expansion. **(D)** Metabolites produced by gut microbiota also play a role in the fate differentiation of HSCs. For example, the gut microbiota metabolite butyrate regulates iron acquisition, which in turn controls HSCs self-renewal and differentiation. In mice that have survived radiation exposure, the gut microbiota metabolite SCFA promotes hematopoietic recovery. (Created with BioRender.com, academic licenses have been granted).

HSCs undergo differentiation in the bone marrow with the involvement of various progenitor cells in either the lymphoid or myeloid lineage. This process is regulated by multiple intrinsic and extrinsic signals. It has been well-established that growth factors, have an impact on hematopoiesis in the bone marrow. Moreover, emerging evidence suggests that gut microbiota also play a role in modulating the ability of HSCs to proliferate and differentiate by influencing the microenvironment of the bone marrow (**Figure 3**).

### 4.1 Gut microbiota influences hematopoiesis by modulating mechanical properties in bone marrow

Studies in mice have demonstrated that the gut microbiota can have an impact on bone morphology and density in the bone marrow (65, 66). Furthermore, gut microbiota influences the rate of bone loss after sex hormone depletion, with germ-free mice being protected against bone loss following ovariectomy (67). It has been observed that germ-free mice exhibit increased bone mass compared to mice with normal microbiomes (68), suggesting a connection between alterations in the gut microbiota and changes in bone microstructure. Antibiotic treatment, which disrupts the gut microbiota, has been associated with changes in bone morphology and density, as well as reduced bending strength and impaired material properties of bone tissue. This disruption is also linked to a decrease in CD20^+^ B and CD3^+^ T cell populations in the spleen (69). The regulation of HSCs is influenced by signals from the bone marrow niche, and patients with osteoporosis, characterized by decreased bone deposition and lower levels of parathyroid hormone, exhibit reduced activity. Thalassemia patients also show reduced HSC resting due to alterations in the characteristics of the bone marrow matrix niche (70). Gut microbiota can disrupt the balance between bone formation and resorption by indirectly affecting the activity of osteoblasts and osteoclasts. Additionally, gut microbiota influences bone metabolism by modulating growth factors, altering bone immune status, and impacting the metabolism of serotonin, cortisol, and sex hormones in mice. The composition of the gut microflora, and consequently bone health, can be altered by probiotics, antibiotics, and diet (71).

Osteocytes play a crucial role as matrix cells in the bone marrow niche. In a recent study, researchers utilized a TLR9^−/−^ C57BL/6 mouse model to demonstrate, for the first time, that the absence of TLR9 in mice leads to trabecular bone loss. This bone loss is attributed to an increase in osteoclastic activity. Further investigations have revealed that chronic systemic inflammation, caused by the altered gut microbiota due to TLR9 changes, is the primary factor contributing to osteoclastic bone loss. Single-cell RNA sequencing (scRNA-seq) analysis has also revealed that HSPCs in TLR9^−/−^ mice show a bias towards the myeloid lineage. This bias is characterized by an upregulation of bone marrow characteristic genes, activation of myeloid-related TFs, and downregulation of lymphoid developmental genes (72).

In mice with obesity induced by a high-fat diet, the alteration of gut microbiota leads to impaired functional niches of the bone marrow microenvironment. Micro-CT analysis of bone structure showed a reduction in bone volume and the number of bone trabeculae in obese mice. Additionally, there was a decrease in LSK cells and a conversion of lymphocytes to myeloid differentiation. These changes were achieved by activating PPARγ2, inhibiting the generation of osteoblasts, and simultaneously strengthening the production of bone marrow adipocytes. The expression of genes in the bone marrow niche, such as Jag-1, SDF-1, and IL-7, was highly suppressed after the high-fat diet (HFD). Furthermore, changes in the microbial community structure were associated with HFD-induced bone marrow changes. Notably, antibiotic treatment was able to rescue the effects of HFD-mediated damage to the bone marrow niche (73). Overall, these findings suggest that manipulating the diversity of gut microbiota could be a potential approach for preventing inflammatory bone loss in the future.

### 4.2 Gut microbiota affects hematopoiesis through inflammatory signals in bone marrow

Inflammation has significant effects on the hematopoietic hierarchy, as it activates and mobilizes effector cells in response to challenges faced by the organism. It also stimulates HSCs and HSPCs to replenish depleted lineage cells, thus restoring homeostasis *in vivo* (74). Recent studies have revealed that microorganisms can indirectly influence the differentiation of HSPCs by releasing inflammatory cytokines through mature immune cells and non-hematopoietic cells during both homeostasis and infection. This process can alter the hematopoietic niche, thereby regulating HSPCs’ homing, differentiation, and proliferation (73, 75).

Infection is a common inflammatory stressor in the hematopoietic system. Following an infection, bone marrow cells decreased, but microbial components and pro-inflammatory cytokines can prompt HSPCs to undergo emergency marrow regeneration (76). Microbial components induce the proliferation and differentiation of HSPCs towards the myeloid lineage, resulting in an increase in the number of myeloid progenitor cells and mature myeloid cells. For example, sepsis induced by cecal ligation and puncture in mice leads to increased bone marrow production, elevated neutrophil counts, and aggravated inflammation (77). Similarly, infection with murine malaria causes an increase in splenic granulocytes and myeloid progenitor cells in the bone marrow (78). *Candida albicans* infection also enhances the production of HSPCs and macrophages (79). Commensal gram-negative bacteria like *Escherichia coli* can expand HSPCs during acute intestinal inflammation and guide them to the inflamed mesenteric lymph nodes via GM-CSFR activation. These HSPCs can potentially differentiate into Ly6C^+^/G^+^ bone marrow cells specialized in intestinal tissue repair (80). At the molecular level, HSCs stimulated by lipopolysaccharide (LPS) undergo chromatin remodeling following a secondary bacterial challenge, which leads them to approach myeloid-specific enhancers and produce more GMP. This indicates that LPS can epigenetically prime HSCs and promote the expansion of myeloid progenitors (81). Different microbial components have other effects as well. LPS selectively induces monocyte and neutrophil differentiation, while CpG DNA promotes monocyte and dendritic cell production (82). During *Escherichia coli* infection, HSCs are significantly reduced, but HSCs in mice treated with antibiotics (ABX) exhibit an increase.

In the analysis of the effects of ABX mice on HSCs production in response to LPS, it was observed that HSCs expansion only occurred in ABX mice (83). After being infected systemically with Listeria monocytogenes, both germ-free and orally antibiotic-treated mice showed an increased pathogen burden and acute mortality. However, when the gut microbiota was reconstituted in germ-free mice, defects in bone marrow production and resistance to *Listeria* monocytogenes were restored (12), suggesting that the gut microbiota plays a role in guiding the development of innate immune cells by promoting hematopoiesis.

### 4.3 Gut microbiota regulates HSCs by altering bone marrow matrix cells

The bone marrow niche consists of various types of cells, including HSCs, MSCs, monocytes, macrophages, and others. These cells, collectively referred to as matrix cells, are capable of recognizing each other as well as HSCs. Through intercellular communication, matrix cells can potentially influence the differentiation and proliferation of HSCs. Recent studies have also revealed that microorganisms can indirectly impact the differentiation of HSCs and HSPCs through interactions with matrix cells, which can alter the hematopoietic niche (73, 75).

MSCs in the bone marrow express multiple pattern recognition receptors and produce inflammatory cytokines when exposed to Toll-like receptor (TLR) agonists (84, 85). The NOD1 ligand from the microbial community induces the production of various hematopoietic cytokines (IL-7, Flt3L, SCF, TPO, and IL-6) in MSCs, contributing to the expansion of the HSPC pool and the maintenance of steady-state hematopoiesis (58). However, the NOD1 ligand alone does not significantly stimulate the proliferation of HSCs, MPPs, or CLPs, nor enhance the expansion of these cells induced by cytokines. Moreover, MSCs from germ-free mice exhibit dysregulated cytokine production and increased culture proliferation, but these abilities normalize when germ-free mice are colonized with microbiota (75). ScRNA-seq reveals that changes in gene expression related to metabolic pathways, HIF-1/inflammatory signaling, and neurodegenerative pathways are associated with the abnormal functionality of MSCs in germ-free mice (75). Recently, lactate derived from the gut has been found to be related to the expression of SCF on MSCs and, along with type I interferons (IFN-1), Stat signaling (86), and iron availability (83), it also regulates hematopoiesis in the bone marrow (87). The gut microbiota stimulates the expansion of macrophages and the quantity and differentiation of GMPs in the bone marrow, facilitating the development of bone marrow cells, maintaining the activity of HSCs and lymphocytes, and promoting hematopoietic homeostasis and host immune responses to pathogens (12).

CX3CR1^+^ monocytes coexist with hematopoietic progenitor cells in the perivascular area. When studying how microbial-derived molecules, such as MDM, migrate to the bone marrow and exert their control over hematopoiesis, it has been found that bacterial DNA reaches the bone marrow through systemic blood circulation and is captured by CX3CR1^+^ monocytes. These monocytes sense the bacterial DNA through TLRs and produce TNF-α, IL-1β, and IL-6, which control the expansion of hematopoietic progenitor cells without affecting the differentiation potential of HSCs (87).

### 4.4 Gut microbiota affects HSCs through metabolites

Microbiota-derived metabolites have been shown to have an impact on HSCs. For instance, lactate, a microbial product, reaches the bone marrow through the bloodstream and stimulates LepR^+^ MSCs surrounding the marrow sinus to secrete SCF, which is crucial for the proliferation of HSCs. This activation of HSCs for hematopoiesis and erythropoiesis occurs in a Gpr81-dependent manner (88). In mice with radiation-induced damage to the hematopoietic system, certain “elite survivors” were found to have a unique gut microbiota composition. Bacterial taxa such as *Lachnospiraceae* and *Enterococcaceae* were associated with post-radiotherapy hematopoietic recovery and gut repair. These bacteria were also more abundant in leukemia patients undergoing radiation therapy. The gut microbiota promotes post-radiation hematopoiesis and intestinal repair by producing microbe-associated MAMPs while reducing pro-inflammatory reactions (89).

Exposure to benzene has been found to hinder the self-renewal and erythroid differentiation of mouse hematopoietic progenitor cells. A study analyzing the gut microbial composition and metabolism revealed that guanidine derived from *Mollicuts_RF39* may play a vital role in the early hematopoietic damage caused by benzene exposure (90). Additionally, the gut microbial metabolite indole-3-propionic acid has been shown to alleviate damage to the hematopoietic system and stomach caused by radiation exposure (91). Zhang et al. also discovered that the gut microbial community regulates macrophages in the bone marrow, influencing the self-renewal and differentiation of HSCs by ensuring iron acquisition in the bone marrow under stress conditions. Another study found that radiation inhibited the activity of HSCs in mice treated with ABX, while ABX treatment led to a significant increase in HSCs in the bone marrow (83). Furthermore, exposure to nanoplastic pollutants, which can enter the bone marrow and affect hematopoiesis, inhibited the renewal and differentiation abilities of HSCs, but supplementation with probiotics was able to reverse the nanoplastic-induced hematopoietic damage by influencing the composition of the gut microbiota and metabolites (92).

With age, the microbiota undergoes changes and there is an increase in gut permeability. In a study on the aging of HSCs, it was observed that transplanting the gut microbiota of young mice into aged mice resulted in the reversal of the gut structure and reshaping of the gut microbial composition and metabolic spectrum in the aged mice. The presence of *Lachnospiraceae* bacteria and tryptophan-related metabolic products was found to promote the recovery of hematopoietic function. This effectively enhanced hematopoietic lineage differentiation in elderly recipient mice, improved the self-renewal ability of HSCs, and enhanced their hematopoietic engraftment ability for short-term-HSCs and long-term-HSCs. Furthermore, it was observed that this intervention could reverse the aging of HSCs (93).

## 5 Gut microbiota influences the development of hematologic malignancies through the bone marrow microenvironment

**Figure 4:**
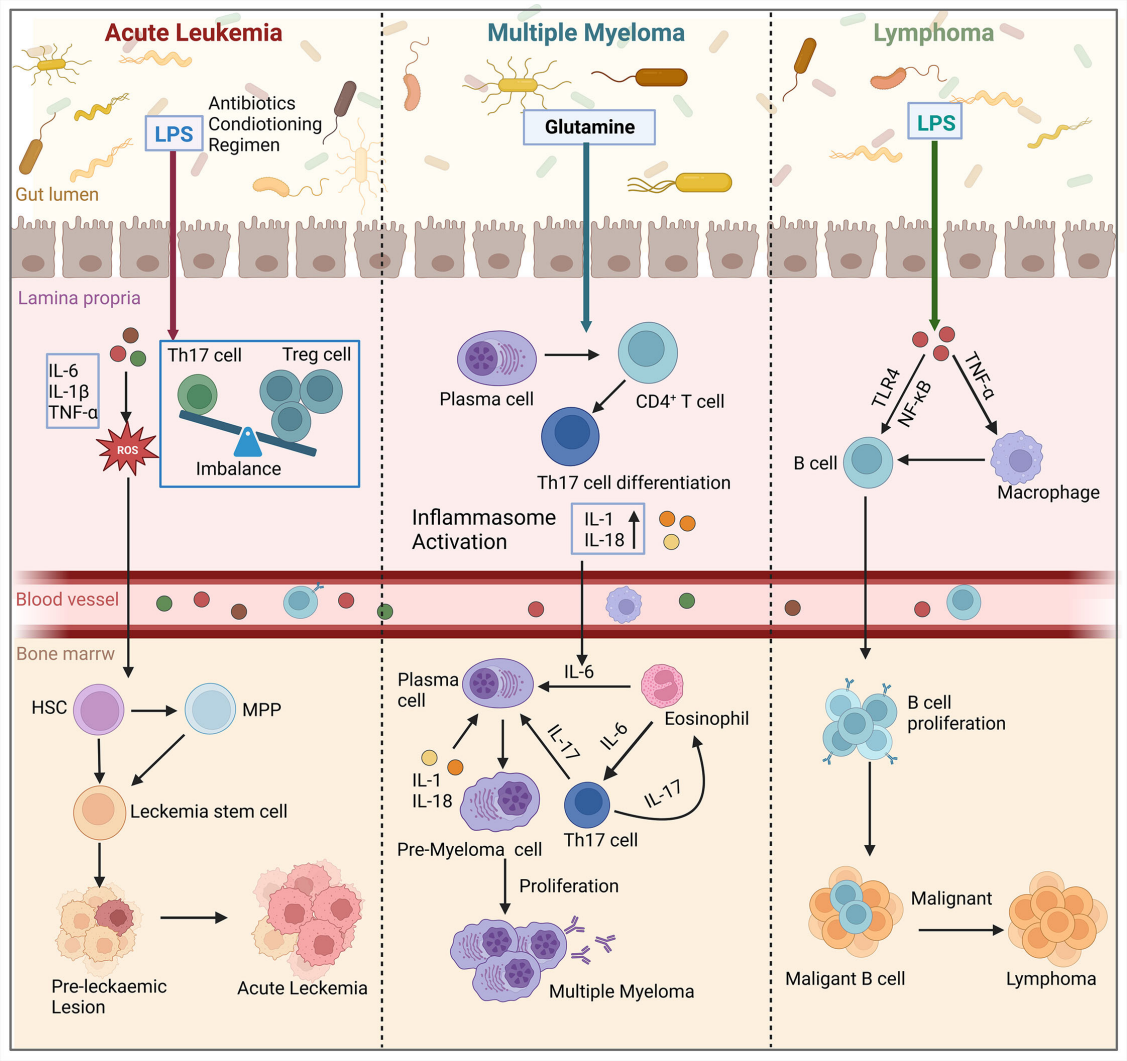
Gut microbiota influence the development of hematologic malignancies (leukemia, multiple myeloma, lymphoma) through the bone marrow microenvironment. In acute leukemia, microbiota dysbiosis leads to an increase in the number of LPS-producing bacteria. This disrupts the intestinal barrier and causes an imbalance of Tregs and Th17 cells in the gut. Additionally, pro-inflammatory factors such as IL-6, IL-1β, and TNF-α are secreted, which activate the production of ROS. This results in oxidative damage and promotes the conversion of HSCs and MPP into leukemic stem cells in the bone marrow, thereby affecting cancer progression. In multiple myeloma, microbial imbalance in mice stimulates the differentiation of plasma cells and CD4^+^ T cells into Th17 cells. Th17 cells migrate to the bone marrow and produce IL-17, which induces plasma cell proliferation and contributes to the progression of multiple myeloma. In lymphoma patients, the presence of LPS in the microbiota interacts with TNF signaling and enhances the NF-κB pathway through TLR4 signaling. Ultimately, this leads to increased survival and proliferation of intestinal B cells, which in turn induces lymphoma in the bone marrow. (Created with BioRender.com, academic licenses have been granted).

The homeostatic balance between HSCs self-renewal and differentiation, which is crucial for replenishing lost blood cells, is regulated by complex interactions between intrinsic and extrinsic factors. Improper regulation of these competing programs can lead to the malignant proliferation of HSCs, resulting in the transformation of these cells into tumor cells and the development of malignant disorders in the blood system. Hematological malignancies arising from hematopoietic abnormalities are highly diverse and associated with a poor prognosis and high mortality rate. The development of these malignancies is influenced by genetic, microenvironmental, and metabolic factors (94). Recently, several studies have demonstrated that gut microbiota play an important role in the development of hematologic malignancies, and that gut microbiota affect the development and progression of hematologic tumors by altering the bone marrow microenvironment (**Figure 4**).

### 5.1 Acute leukemia

Leukemia is a condition where abnormal hematopoietic stem cells proliferate uncontrollably, leading to the destruction of normal bone marrow function and bone marrow failure. This results in the uncontrolled growth of malignant clones (95). Leukemic cells can be categorized into acute and chronic types based on their level of differentiation and natural progression. Acute leukemia (AL) originates from primitive hematopoietic stem cells and early naïve cells, progresses rapidly, and has a relatively short course. It can further be classified into acute lymphoblastic leukemia (ALL) and acute myeloid leukemia (AML) based on the cell type (96). The interaction between leukemic cells and the microenvironment plays a crucial role in the development of ALL. Leukemic cells remodel the bone marrow ecological niche, creating an immunosuppressive microenvironment that promotes leukemic progression. Studies have shown that this remodeling leads to impaired function of various immune cells, such as NK cells, T cells, and macrophages (97). Additionally, there is an increase in immunosuppressive agents like Tregs and granulocyte-myeloid-derived suppressor cells (G-MDSCs). Tregs secrete inhibitory cytokines that suppress the cytotoxic activity of T cells and reduce macrophage phagocytosis. On the other hand, G-MDSCs produce reactive oxygen species (ROS) and inhibit NK cell activity. In the bone marrow ecological niche, MSCs also contribute to leukemic cell growth by secreting chemokines, NF-κB, and metabolites (98). Gut microbiota plays a role in the development and progression of leukemia by influencing the bone marrow microenvironment. However, an intact microbiota can prevent leukemia growth. The microbiota produces metabolites, including SCFAs, which help maintain the intestinal epithelial barrier, suppress inflammatory cytokines, and control host immunity through epigenetic changes. SCFAs inhibits histone deacetylase activity, promotes the differentiation of Tregs, and maintains a balance between Tregs and Th17 cells (97). When the microbial ecology is dysregulated, leukemia progression can occur. Factors such as antibiotics, pretreatment regimens, changes in diet or medications can lead to a loss of beneficial bacteria and an increase in pathogenic bacteria. An increase in LPS-producing bacteria can cause mucositis, disruption of the mucus layer, and impairment of the intestinal barrier (99, 100). This leads to the leakage of LPS into the bloodstream, promoting leukemia progression. Consequently, pathogens can invade the lamina propria and activate the immune response, resulting in an imbalance of Tregs and Th17 cells and the secretion of proinflammatory cytokines like IL-1β, IL-6, and TNF-α. These cytokines, in turn, stimulate the production of ROS, nitrogen, and sulfur, causing oxidative damage and further inducing leukemia progression (101).

### 5.2 Multiple myeloma

Multiple Myeloma (MM) is the second most common hematologic malignancy characterized by the accumulation of malignant plasma cells in the bone marrow. The development of MM is a complex process influenced by genetic and environmental factors, resulting in various pathomechanisms. MM development is closely associated with changes in its bone marrow microenvironment, such as chronic antigenic B-cell stimulation, inflammation, and immune modulation (102). Patients with MM and mouse models of MM commonly exhibit elevated levels of inflammatory cytokines, including TNF-α and IL-6, which are linked to disease onset, progression, symptom burden, and prognosis (103, 104). Inflammation can create an environment that promotes the survival of tumorigenic HSCs (105). Mutant HSPCs have shown resistance to inflammation, possibly through apoptosis induction, inactivity, or enhanced self-renewal and differentiation of HSCs (106). Moreover, the mutant cells themselves can produce inflammatory cytokines, particularly TNF-α, and stimulate the production of inflammatory cytokines by surrounding normal cells (107, 108).

The composition of the microbiome and its metabolites also have an impact on the bone marrow microenvironment, influencing the development of myeloma. In Rag-1-deficient mice, dysregulation of the microbiota leads to a decrease in the production of HSCs and HSPCs, resulting in a reduction in lymphocytes. Dysbiosis of the microbiota has also been associated with a decrease in the production of SCF by the microbiota, which can lead to lower levels of SCFA. This, in turn, can increase the activation of inflammatory mediators like IL-6 and the NF-κB pathway, potentially contributing to the progression of myeloma. Moreover, an increase in the nitrogen-cycling microbiota in the intestinal lumen may promote glutamine synthesis and contribute to the progression of myeloma (109). In a transgenic mouse model, specific bacteria like *Prevotella heparinolytica* can promote the differentiation of Th17 cells, which then migrate to the bone marrow and produce IL-6. This IL-6 induces Stat3 phosphorylation in mouse plasma cells, activating eosinophils and further promoting the release of IL-6, thus contributing to inflammation-mediated progression of MM (110).

### 5.3 Lymphoma

Lymphomas are malignant tumors that arise from the abnormal proliferation and differentiation of lymphocytes during an immune response. These tumors can develop in various parts of the body and are characterized by painless enlargement of lymph nodes. Localized masses are a common clinical manifestation, often accompanied by symptoms of organ compression. Lymphomas are classified into Hodgkin’s lymphoma and non-Hodgkin’s lymphoma based on histopathologic features (111). In animal models, the gut microbiota has been identified as a potential contributing factor to lymphomagenesis (112). Tegla et al. conducted a study on lymphoma patients and found an increased presence of *Staphylococcus* spp., a genus of bacteria, compared to healthy individuals. The *Staphylococcus* spp. directly influences antigen presentation, T-cell clonal expansion, and the production of pro-inflammatory cytokines, thereby promoting disease progression in lymphoma (113). Another study analyzed the gut microbiota of lymphoma patients and identified a distinct microbial signature (114). They observed a significant decrease in commensal microorganisms, particularly the butyrate-producing *Eubacterium rectum*, in lymphoma patients. Furthermore, when the researchers transferred *Eubacterium rectum*-deficient microbiota from lymphoma patients into mice, it triggered inflammation and TNF production. The presence of LPS in the microbiota of lymphoma patients interacted with TNF signaling and enhanced the NF-κB pathway through MyD88-dependent TLR4 signaling, leading to increased survival and proliferation of intestinal B cells. In the gastrointestinal tract, defective intestinal microbiota of *Eubacterium rectum* stimulates B cells by releasing TNF, which in turn sensitizes B cells to LPS. These extracellular substances can bind to membrane TNF and TLR4, activating NF-κB signaling through a MyD88-dependent mechanism as an extrinsic pathway. These discoveries provide insights into the mechanisms behind inflammation-associated lymphoma and suggest the possibility of targeting the gut microbiota for therapeutic interventions.

## 6 Concluding and perspectives

Mature HSCs are primarily located in the bone marrow niche, where the bone marrow microenvironment controls their self-renewal and fate differentiation. There is growing evidence suggesting that the gut microbiota influences HSCs within the bone marrow and plays a crucial role in determining their fate. However, the specific mechanisms by which the gut microbiota regulates HSCs fate through the bone marrow microenvironment are still not well understood. In the *in vivo* bone marrow niche, the gut microbiota can impact HSCs fate differentiation and proliferation by affecting various factors such as bone marrow mechanical properties, inflammatory signals, bone marrow stromal cells, and metabolites. During the development of hematologic malignancies, HSCs can alter the microenvironment, thereby influencing the disease process. The gut microbiota is essential in hematopoiesis and cancer progression through the bone marrow microenvironment. Future studies will enhance our understanding of how the microbiota regulates the bone marrow microenvironment during hematopoiesis. Hopefully, this knowledge will enable us to develop strategies that leverage the gut microbiota to support healthy hematopoiesis in patients with disrupted gut ecology and improve therapeutic approaches for hematopoiesis-related diseases.

## Author contributions

XL: Writing – original draft, Writing – review & editing. HZ: Visualization, Writing – original draft. GS: Supervision, Writing – review & editing. XZ: Supervision, Writing – review & editing. JC: Writing – review & editing. QL: Writing – review & editing. ZT: Writing – review & editing. HY: Writing – original draft, Writing – review & editing.

## Glossary

**Table:**

HSCs	Hematopoietic stem cells
SCFAs	Short-chain fatty acids
MPPs	Multipotent progenitors
HSPCs	Hematopoietic stem and progenitor cells
CMPs	Common myeloid progenitors
CLPs	Common lymphoid progenitors
GMPs	Granulocyte-macrophage progenitors
MEPs	Megakaryocyte-erythrocyte progenitors
MSCs	Mesenchymal stem cells
CAR	CXCL12-abundant reticular
Tregs	Regulatory T cells
SCF	Stem cell factor
TPO	Thrombopoietin
3D	Three-dimensional
2D	Two-dimensional
OPN	Osteopontin
G-CSF	Granulocyte colony-stimulating factor
IFN	Interferon
TFs	Transcription factors
MAMPs	Microbe-associated molecular patterns
TLRs	Toll-like receptors
NOD	Nucleotide-binding oligomerization domain
Myd88	Myeloid differentiation primary response 88
ScRNA-seq	Single-cell RNA sequencing
HFD	High-fat diet
ABX	Antibiotics
G-MDSCs	Granulocyte-myeloid-derived suppressor cells
ROS	Reactive oxygen species
LPS	Lipopolysaccharide
MM	Multiple myeloma
IFN-1	Type I interferons
AL	Acute leukemia
ALL	Acute lymphoblastic leukemia
AML	Acute myeloid leukemia
